# Mitochondrial DNA affects metabolome, lipidome, and proteome in a sex specific manner in old heterogenous rats

**DOI:** 10.1093/geroni/igaf122.1926

**Published:** 2025-12-31

**Authors:** Hoang Van Nguyen, Chloe Fender, Manuel Garcia-Jaramillo, Ahsan Nagib, Norman Hord, Steven Austad, Arlan Richardson, Michael Stout

**Affiliations:** The University of Oklahoma Health Sciences, Oklahoma City, Oklahoma, United States; Oregon State University, Corvallis, Oregon, United States; Oregon State University, Corvallis, Oregon, United States; The University of Oklahoma, Norman, Oklahoma, United States; Oklahoma State University, Stillwater, Oklahoma, United States; The University of Alabama at Birmingham, Birmingham, Alabama, United States; The University of Oklahoma Health Sciences Center, Oklahoma City, Oklahoma, United States; Oklahoma Medical Research Foundation, Oklahoma City, Oklahoma, United States

## Abstract

The effect of mitochondria-haplotype (mt-haplotype) on aging was studied using a unique rat model (OKC-HETB/W), which has a heterogenous nuclear background and mitochondria from either Brown Norway (B-haplotype) or Wistar Kyoto (W-haplotype) rats that differ in 94 nucleotides. The impact of mt-haplotype on aging was studied using an unbiased multi-omics approach to analyze the plasma from 9- and 26-month-old male and female OKC-HETB/W rats. Of the 280 metabolites, 961 lipids, and 230 proteins that changed significantly with age, 50% to 80% were mt-haplotype specific, occurring in either OKC-HETB or OKC-HETW rats. Interesting, the majority (78-93%) of the changes that were mt-haplotype specific were also sex specific, occurring in either male or female rats but not both. Our data are the first to show the potential importance of mt-haplotype in aging and that this mt-haplotype difference is largely sex dependent.

